# Household livelihood, diet, and nutritional status of adolescent schoolchildren in Kuyu District, North Shewa, Oromia, Ethiopia

**DOI:** 10.1017/jns.2025.10019

**Published:** 2025-10-09

**Authors:** Kassahun Ketema, Aregash Samuel, Mogessie Ashenafi

**Affiliations:** 1 Centre for Food Security Studies, College of Development Studies, Addis Ababa University, Addis Ababa, Ethiopia; 2 Nutrition, Environmental Health and Non-communicable Disease Research Directorate, EPHI, Addis Ababa, Ethiopia

**Keywords:** Adolescent boys and girls, Anthropometry, Diet diversity, Livelihood, FANTA, Food and Nutrition Technical Assistance III Project, WHO, World Health Organization, SPSS, Statistical Package for the Social Sciences, BMI, Body Mass Index, SD, Standard Deviation, HAZ, Height for Age z-score, BAZ, Body Mass Index for Age, VIF, Variance Inflation Factor, CI, confidence intervals, AOR, Adjusted odds ratios, CFSS/CoDS, Center for Food Security Studies/College of Development Studies

## Abstract

Adolescents from low-income households are at increased risk of growth failures due to inadequate food intake. This cross-sectional study assessed dietary practices and nutritional status according to FANTA measurement standards. Among 610 randomly selected adolescents attending public primary schools in rural and semi-urban Kuyu district. Dietary diversity and anthropometric measurements (height, weight, and Body Mass Index) were collected and analysed using SPSS version 26 and WHO Anthro Plus software. The study population included 36% females and 69% semi-urban residents. Dietary analysis revealed that most adolescents consumed two or fewer daily meals, primarily cereals and legumes. Over 90% of the households consumed less than four food groups during the 7-day recall period. The anthropometric assessment showed significant undernutrition: 19% of early adolescent girls and 34% of late adolescent boys were underweight; 27.5% were stunted; 8% and 5.9% had moderate and severe undernutrition, respectively; and 13.8% exhibited thinness, with boys more affected (35%) than girls (10%). Additionally, 7% were overweight, and 64% presented single, double, or triple growth failures. Regression analysis showed that Children in female-headed households had 1.7 times higher odds of stunting, adolescent girls had 1.8 times higher odds of thinness, late adolescents had 70% lower odds of being overweight, and adolescents from households with off-farm activities had 4.5 times higher odds of being overweight. Inadequate meal frequency and limited dietary diversity contribute to the high prevalence of undernutrition among Kuyu district adolescents. A school feeding programme is strongly recommended.

Undernutrition remains a pervasive public health crisis in low- and middle-income countries (LMICs), disproportionately impacting vulnerable populations, particularly adolescents.^(1–3)^ Despite global efforts to combat food insecurity and malnutrition, progress is uneven, and significant challenges persist, especially in regions grappling with adverse environmental and socioeconomic conditions. The imperative to address undernutrition is underscored by its direct relevance to achieving Sustainable Development Goals (SDGs) 2 (Zero Hunger) and 3 (Good Health and Well-being) by 2030.^(4)^

Adolescence represents a critical developmental phase, requiring adequate nutrition for optimal physical growth, cognitive development, and long-term health. However, in LMICs, food insecurity frequently compromises the quality and quantity of adolescents’ nutritional intake,^(3)^ leading to various forms of undernutrition.^(5)^ This vulnerability results in immediate adverse health outcomes and long-term risks, including increased susceptibility to overweight/obesity due to poor dietary patterns and micronutrient deficiencies.^(6)^ Furthermore, food insecurity significantly impacts educational attainment, contributing to absenteeism, reduced school performance, and cognitive impairment,^(7)^ thus perpetuating cycles of poverty and poor health.

Despite its agricultural potential, Kuyu district, Ethiopia, faces acute challenges, including recurrent crop failures, reliance on food aid, and high poverty rates, all contributing to inadequate calorie intake and a deteriorating nutritional situation. Notably, the absence of school feeding programmes within Kuyu exacerbates this vulnerability. Critically, data on adolescent nutrition in Kuyu is severely lacking, unlike in other regions of Ethiopia. This knowledge gap hinders the development of targeted interventions and evidence-based policies.

Therefore, this study is essential to address this critical knowledge gap. The focus of the research question is to investigate the dietary patterns, diversity, and nutritional status of adolescents in Kuyu, focusing on the prevalence, drivers, consequences, and potential solutions for undernutrition, thereby generating crucial empirical evidence. The findings provide actionable insights into developing targeted interventions, ultimately contributing to the achievement of SDGs 2 and 3 and improving the well-being of this highly vulnerable population. This study is not only timely but imperative to break the cycle of undernutrition and its associated consequences in Kuyu district.

## Materials and methods

### Study area

This study was conducted in the Kuyu district, located in the North Shewa Zone of the Oromia regional state of Ethiopia. Situated 156 kilometres north of Addis Ababa, the district spans 950.8 square kilometres, ranking as the fourth largest in the zone. The Kuyu district comprises 23 rural and four semi-urban kebeles, including two in Garba Guracha, one in Bucho Town, and one in Biriti Town. The projected population for 2023 was 187,146, with 50.5% of the population being female.^(8)^ The district houses 63 schools, consisting of 41 primary (grades 1–8) and 22 secondary (grades 9–12) institutions. In 2022, the total number of adolescents attending grades 1–8 was 21,423.^(9)^

### Study design and period

A cross-sectional study design was utilised to collect baseline data from adolescent schoolchildren and their households. Schools were stratified into rural and semi-urban categories, and data were collected from randomly selected institutions. The study was conducted from May 2 to May 28, 2022.

### Source and study population

The source population comprised all primary schools and their enrolled adolescents in the Kuyu district. The study population consisted of randomly selected primary schools and their adolescent schoolchildren. Those older than 19 (considered young adults) and those with physical anomalies that could affect anthropometric measurements, such as weight and height, were excluded.

### Sample size determination and sampling procedures

The sample size was calculated using the single population proportion formula,^(10)^ with a 95% confidence level and a 5% margin of error.

The formula used was:

n = (Zα/2)2×p×(1−p)/d2

where:

Zα/2 = 1.96 (standard normal deviation at 95% confidence interval)

p = 0.434 (proportion of malnutrition among public primary school children in Addis Ababa, as per Destaw et al.^(11)^

d = 0.05 (margin of error)

Substituting the values into the formula:

n = (1.96)2×0.434×(1−0.434)/(0.05)2

n = 377

To account for a potential 10% non-response rate (37 individuals) and a design effect of 1.5, the initial sample size was adjusted. The adjusted sample size was calculated as follows:

Adjusted sample size = (calculated sample size + non-response adjustment) × Design effect

Adjusted sample size = (377 + 37) × 1.5

Adjusted sample size = 414 × 1.5

Adjusted sample size = 621

Therefore, a final sample size of 621 was considered for this study. Data were collected from adolescent schoolchildren and their respective mothers or caregivers.

### Sampling technique/procedure

**Fig. 1.**
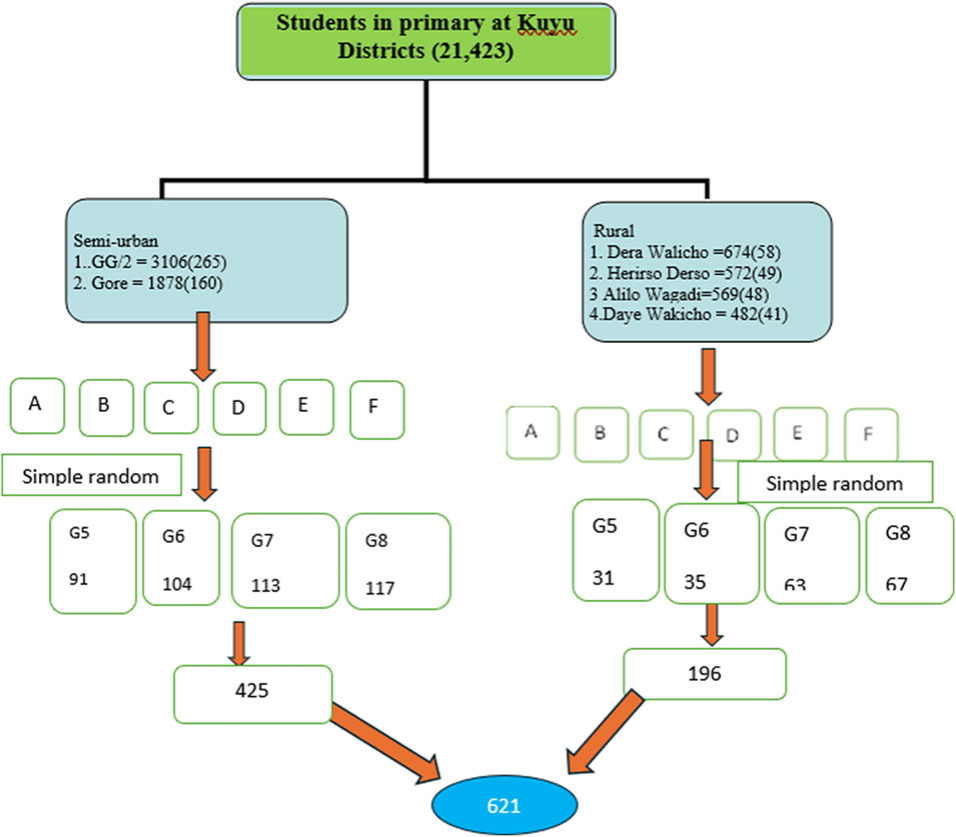
The schematic representation of sampling on study subjects randomly in each step.

A multi-stage sampling technique was used to select representative samples from each semi-urban and rural school in Kuyu district. First, a list of all the schools in the district was compiled. From this list, six schools were randomly selected: two semi-urban and four rural schools, with the selection proportional to the number of schoolchildren in each school (Figure 1).

Within the selected schools, adolescent schoolchildren in grades five to eight were randomly selected for their ability to understand and respond to questionnaires. Proportional allocation was used to determine the sample size for each schoolchild, grade, and section. Mothers/caregivers of the selected adolescents were also interviewed.

### Data collection tools and procedure

Data collection was conducted by health professionals from Kuyu District Hospital who received training that covered the study’s objectives, standardised data collection procedures, and ethical considerations, including obtaining informed consent. Special emphasis was placed on techniques to minimise recall bias during interviews. The principal investigator provided direct supervision throughout the data collection phase, ensuring strict adherence to the study protocol. This oversight included conducting spot checks and verifying data quality to maintain accuracy.

To minimise respondent bias, all survey instruments were rigorously pre-tested and piloted before the main data collection began. Structured self-reporting questionnaires were then used to gather information from sampled households on socio-demographic characteristics, household livelihood strategies, and dietary diversity. Concurrently, anthropometric measurements were systematically taken from the sampled students.

### Assessment of household livelihood

A livelihood system is the total combination of activities undertaken by a typical household to ensure a living. Household livelihood data were collected through self-reporting questionnaires that focused on land ownership, landholding size, crop yield, and the adoption of agricultural technologies, including the use of improved seeds, fertilisers, and irrigation.

### Assessment of household dietary diversity

Household dietary diversity is the number of food groups consumed by a household over a given reference period and is an important indicator of food security. Household dietary diversity was assessed using a 7-day food recall, following Swindale and Bilinsky,^(12)^ using a 12-food group questionnaire. The food groups included: cereals, starches, green leafy vegetables, other vegetables, fish/seafood, meat, eggs, nuts/legumes, fruits/juice, oils/fats, sauces, and beverages/biscuits/sweets. Participants were asked whether their household had consumed any food item from each of these groups in the seven days preceding the survey. For mixed dishes, detailed ingredient information was collected. Responses for each food group were recorded as ‘yes’ or ‘no’. A score of 1 was assigned for each food group with a ‘yes’ response (indicating consumption of at least one item from that group in the recall period), and 0 for a ‘no’ response. These scores were summed to generate a household dietary diversity score for each household, following FAO guidelines. As in Kolliesuah et al.^(13)^, consumption of ≤ 5 food groups in the previous 24 hours prior to the survey was considered low in dietary diversity, 6–8 food groups as medium and ≥ 9 as high dietary diversity score using the FAO 12 food groups.^(14)^

### Anthropometric measurements

Anthropometric measurements are used to assess body size and body composition. These measurements are simple, safe, and non-invasive, providing information on past exposure. The height and Weight of adolescent schoolchildren were measured in schools using standardised techniques following Gibson.^(15)^ If two or more adolescent schoolchildren came from the same household, one was selected by lottery. Weight was measured with a SECA 786 digital weighing scale, and height was measured with a portable measuring Stadiometer. All participants removed their shoes and head coverings and were asked to place their heels at the base of the stadiometer prior to being measured. Each height and weight measurement was taken twice, and the digital scale was recalibrated before each measurement.

If a child has a low height-for-age, i.e. a Z-score below two standard deviations of the reference population mean (−2 Z-score), such a child is categorised as ‘stunted’. Similarly, a low weight-for-age is diagnostic of an ‘underweight’ child, while a low weight-for-height is indicative of ‘thinness’.^(16)^ A Body Mass Index (BMI) greater than the 85th percentile in adolescence is indicative of a risk of being overweight. BMI-for-age was calculated as: BMI = weight (kg)/height (m)^2^. Using BMI-for-age z-score,^(16)^ the results were categorised as normal, underweight, or overweight, according to Sethi et al.^(17)^ and Cashin & Oot.^(16)^ All measurements were conducted by trained health professionals.

The nutritional status of an individual is the result of the nutritional intake received and the nutritional demands, allowing for the utilisation of nutrients to maintain reserves and compensate for losses. Nutritional status was categorised using WHO AnthroPlus international growth reference, expressed as standard deviations (SD) from the median^(18)^. Undernutrition, a composite outcome, was defined as having stunting (Height for Age (HAZ) < −2 SD), thinness (weight for height < −2 SD), or BMI for Age (BAZ, < −2 SD), or all. Overweight was defined as BAZ > +1 SD.

### Data processing and analysis

Questionnaire data, following coding and quality checks, were analysed using SPSS version 26. Anthropometric data were analysed with WHO Anthro Plus. Continuous data were summarised using means and standard deviations (SD), medians, and interquartile ranges, while categorical data were described using frequency distributions. Multicollinearity was assessed, with variables exceeding a Variance Inflation Factor (VIF) of 10 excluded.

Bivariate and multivariate regression models were fitted. Variables with p < 0.25 in bivariate analysis were included in the multivariate model, after assumption checks. Statistical significance in multivariate analysis was defined as p < 0.05. Adjusted odds ratios (AOR) with 95% confidence intervals (CI) were used to report the strength and significance of associations.

### Ethical Consideration

Ethical approval was obtained from the Ethical Review Board of the College of Development Studies, Addis Ababa University (Ref. N0: CFSS/CoDS/531/2013). Relevant authorities at the zone, district, and school levels also granted permission to conduct the study. The purpose and nature of the study were explained to the parents of adolescent schoolchildren, and informed verbal consent was obtained from 18-year-old adolescent schoolchildren and household heads of children under 18 prior to their children’s participation. All participants were assured of their right to withdraw at any time. Confidentiality of information obtained from them and their anonymity were strictly maintained throughout the study.

## Results

### Socio-demographic characteristics

A total of 610 adolescent schoolchildren (98.2% response rate, mean age, 14.3 years; range 10-18 years) participated. Despite Ethiopia’s official primary school entry age of 7, approximately 54% of children, particularly in rural areas, experience delayed enrolment. The sample comprised predominantly semi-urban residents, with a third being female (Table 1). All schools were public institutions. A small proportion of adolescents reported disabilities, mainly physical. Most reported minimal school absenteeism and illness. Academic performance averaged 68% (range 47–98%).

**Table 1.** Socio-demographic characteristics of households and school children (n = 610) in Kuyu district

Households	Variable	Category	Frequency (%)
	Sex	Female	222 (36.4)
		Male	388 (63.6)
	Residence	Rural	192 (31.5)
		Semi-urban	418 (68.5)
	Age	10–14	441 (67.9)
		15–19	196 (32.1)
	Kind of disability	Sight	7 (1.1)
		Physical	13 (2.1)
		Hearing	5 (0.8)
	Experienced illness	Diarrhoea	43 (7.0)
		Febrile illness	16 (2.6)
		Others	4 (0.7)
Schoolchildren	Sex	Male	259 (42.5)
		Female	351 (57.5)
	Age	< 30 years	394 (64.6)
		> 30 years	216 (35.4)
	Residence	Rural	335 (54.9)
		Semi-urban	275 (45.1)
	Educational status	Can not read and write	219 (35.9)
		Grades 1 to 8	357 (58.5)
		Secondary and post-secondary	34 (5.6)
	Marital status	Married	449 (73.6)
		Divorced, separated, single	161 (26.4)
	Occupation	Farmer	492 (80.7)
	Family size	Employee	75 (12.3)
		Others (daily labour)	43 (7.0)
		< Four	251 (41.1)
		> Five	359 (58.9)

Household respondents (n = 610) were primarily married females, aged 30 or younger, residing in rural areas, and of Oromo ethnicity (Table 1). Most had basic literacy or elementary education and were farmers. A minority reported family sizes of five or more. Although 80% of the respondents were farmers, 40% cultivated rented land with varying crop yields (mean, 135.3 kg). Adoption of improved agricultural technologies was low, and 12.5% (n = 76) reported off-farm income (Table 2).

**Table 2.** Livelihood of households in Kuyu district, North Shewa Zone, Oromia, Ethiopia, 2022 (n = 369)

Variables	Category	Frequency (%)
Have land ownership	Semi-urban	120 (32.5)
	Rural	249 (67.5)
	< 100	170 (46.1)
Average crop harvest (kg)	100–200	137 (37.1)
	201–300	43 (11.7)
	> 301	19 (51.1)
Landholding (ha)	< 1	303 (82.1)
	>= 1	66 (17.9)
Average quintals per ha		1.71±1.8
Use improved seeds		197 (53.4)
Use fertilisers		220 (59.6)
Use Irrigation		45 (7.4)
Have off-farm income		76 (12.5)

### Dietary patterns and diversity

Breakfast consumption was low, while lunch was nearly universal, and supper was consumed by half the adolescents (Table 3). Rural adolescents reported higher rates of breakfast and dinner consumption. Dietary diversity was poor, with over 90% of households offering fewer than four food groups. Cereals and legumes were consistently consumed, while root crops and animal protein were infrequently reported. Fruit consumption was also low, and no religious dietary restrictions were observed.

**Table 3.** Dietary diversity practice of households in Kuyu district, North Showa Zone, Oromia, Ethiopia, 2022

Variables	Category	Frequency (%)		
		Semi-urban	Rural	Total
		(n = 275)	(n = 335)	(n = 610)
Break fast		47 (17.1)	80 (23.9)	127 (20.8)
Lunch		272 (98.9)	328 (97.9)	600 (98.4)
Dinner		129 (46.9)	178 (53.1)	307 (50.3)
Frequency of eating per day	< 3 times	223 (81.1)	257 (82.1)	480 (78.7)
	>= 3 times	52 (18.9)	78 (23.3)	130 (21.3)
Cereals		275 (100)	335 (100)	610 (100)
Legumes		275 (100)	335 (100)	610 (100)
Roots and tubers		39 (10)	67 (20)	106 (17.4)
Vegetables (kale, cabbage, pepper)		143 (52)	218 (65.1)	360 (59.0)
Fruits (Orange, lemon)		24 (8.7)	44 (13.1)	68 (11.1)
Flesh meat		12 (4.4)	31 (9.3)	41 (7.1)
Eggs		21 (7.6)	63 (18.8)	85 (13.9)
Fish and sea food		4 (1.6)	4 (1.2)	8 (1.3)
Milk and its products		93 (33.8)	47 (14)	140 (23.0)
Oils and fats		81 (29.6)	141 (42.1)	222 (36.4)
Sugar and honey(sweets)		61 (22.2)	90 (26.9)	148 (24.3)
Miscellaneous (condiments, beverage)		75 (27.3)	110 (32.8)	185 (30.2)
DDS	> 4food group	13 (4.7)	28 (8.4)	41 (6.7)
	< 4food group	262 (95.3)	307 (91.6)	569 (93.3)

### Anthropometric measurements

Anthropometric assessments, using height-for-age, weight-for-height, and weight-for-age z-scores, classified adolescents as normal, wasted, stunted, or underweight. The overall prevalence of thinness was low, with a slight elevation in early adolescent girls (Table 4). While thinness remained low in late adolescence for both sexes, the prevalence of underweight was notably higher in late adolescent boys compared to girls and to younger adolescents overall. The total prevalence of underweight was 25.4%.

**Table 4.** Prevalence of underweight and wasting in adolescent schoolchildren and adolescents in the study Woreda

				Weight-for-age		Weight-for-height		Height-for-age		
Age group	Sex	Number	Median Weight (kg)	Underweight –2 z-score	Normal	Thinness –2 z-score	Normal	Median height (cm)	Stunted –2 z-score	Normal
				n (%)	n (%)	n (%)	n (%)		n (%)	n (%)
10–14	F	62	40	12 (19.4)	50 (80.6)	12 (19.4)	50 (80.6)	146	20 (32.0)	42 (68.0)
	M	107	40	19 (17.8)	88 (82.2)	14 (13.1)	93 (86.9)	148	26 (24.3)	81 (73.7)
	Both	169	40	31 (18.3)	138 (81.7)	26 (15.4)	143 (84.6)	148	46 (27.2)	123 (72.8)
15–19	F	160	42.5	30 (18.8)	130 (81.2)	21 (13.1)	139 (86.9)	154	46 (28.8)	114 (71.3)
	M	281	44	94 (33.5)	187 (66.5)	37 (13.2)	244 (86.8)	153	76 (27.0)	205 (73.0)
	Both	441	43	124 (28.1)	317 (71.9)	58 (13.2)	383 (86.8)	153	122 (27.8)	319 (72.2)
All		610		155 (25.4)	455 (71.3)	84 (13.8)	526 (86.2)		168 (27.5)	442 (72.5)

Stunting, which affects 27.5% of the population (Table 4), showed age and sex variations. Early adolescent girls have higher stunting rates than boys, but this trend reverses in late adolescence, when boys exhibit a higher prevalence.

Using BMI-for-age (Table 5), boys displayed significantly higher rates of moderate thinness (35%) than girls (10%). Severe thinness, though less common, also disproportionately affected boys. The prevalence of overweight remained below 8% across all age groups. In younger adolescents (10–14 years), boys were more likely to be overweight, while girls showed a higher prevalence in older adolescents (15–19 years).

**Table 5.** Body mass index measurement of school-going adolescents in the study Woreda

			Body Mass Index (BMI)			
Age group (years)	Sex	Number	Normal –1 to 1 z-score	Moderate thinness < –2 z-score	Severe thinness < –3 z-score	Over weight > 1SD
10–14	F	114	96 (84.2%)	13 (11.4%)	3 (2.6%)	2 (1.8%)
	M	236	141 (59.7%)	60 (25.4%)	18 (7.6%)	17 (7.2%)
	Both	350	237 (67.6%)	73 (20.9%)	21 (6.0%)	19 (5.4%)
15–19	F	108	83 (76.9%)	8 (7.4%)	5 (4.6%)	12 (11.1%)
	M	152	120 (79.1%)	14 (9.2%)	13 (8.6%)	5 (3.3%)
	Both	260	203 (78.1%)	22 (8.5%)	18 (6.9%)	17 (6.5%)
10–19	F	222	179 (80.6%)	21 (9.5%)	8 (3.6%)	14 (6.3%)
	M	388	261 (67.3%)	74 (19.1%)	31 (8.0%)	22 (5.7%)
	Both	610	440 (72.1%)	95 (14.6%)	39 (6.4%)	36 (5.9%)

Less than half of the adolescents were free from any growth failure, with a majority of those being boys (Table 6). Younger adolescents (10–14 years) experienced a higher proportion of growth failures than older adolescents (15–18 years) in both sexes. The most frequent multiple growth failure was stunting combined with overweight. Nearly half of the adolescents exhibited a single growth failure, while a small subset had two or three concurrent growth failures.

**Table 6.** Composite index of anthropometric failure in adolescent schoolchildren in Kuyu District

	CIAF		Girls N (%)					Boys N (%)				Total
			Age group					Age group				N (%)
			10–14		10–14		10–14	10–14		15–19	10–19	10–19
A	Without growth failure		45 (31.7)	126 (34.2)		171 (33.5)		28 (33.3)	82 (42.3)		110 (39.6)	281 (35.6)
B	Thinness only		7 (4.9)	24 (6.5)		31 (6.1)		4 (4.8)	14 (7.2)		18 (6.5)	49 (6.1)
C	Thinness and underweight		1 (0.7)	11 (3.0)		12 (2.4)		2 (2.4)	2 (1.0)		4 (1.4)	16 (2.0)
D	Stunting, thinness, and underweight		–	8 (2.2)		8 (1.6)		2 (2.4)	2 (1.0)		4 (1.4)	12 (1.4)
E	Thinness and stunting		3 (2.1)	14 (3.8)		17 (3.3)		2 (2.4)	6 (3.2)		8 (2.8)	25 (3.3)
F	Stunting only		30 (21.1)	79 (21.5)		109 (21.4)		15 (17.9)	42 (21.7)		57 (20.5)	166 (21.1)
G	Excess weight only		16 (11.3)	20 (5.4)		36 (7.1)		8 (9.5)	10 (5.2)		18 (6.5)	54 (7.4)
H	Stunting and overweight		24 (16.9)	37 (10.1)		61 (12.0)		13 (15.5)	18 (9.3)		31 (11.2)	92 (11.5)
Y	Underweight only		16 (11.3)	49 (13.3)		65 (12.7)		10 (11.9)	18 (9.3)		28 (10.1)	93 (11.9)
Total			142	368		510		84	194		278	788
CIAF		N	97	242		339		56	112		168	507
		%	68.3	65.8		66.5		66.7	57.7		60.4	64.3

### Factors associated with undernutrition

Logistic regression revealed that stunting was 1.7 times more likely in children from female-headed households. Girls were 1.8 times more likely to be thin, and late adolescents were 70% less likely to be overweight. Adolescents from households with off-farm income were 4.5 times more likely to be overweight (Table 7).

**Table 7.** Binary and multiple-variable logistic regression of risk factors for malnutrition among schoolchildren in Kuyu district, North Shewa Zone, Oromia, Ethiopia, 2022

Variables	Category	Frequency (%)		COR (95% CI)	AOR (95% CI)
		Stunting			
		No	Yes		
HH Sex	Male	206 (79.5)	53 (20.5)	1	1
	Female	236 (67.2)	115 (32.8)	1.9 (1.30, 2.75)	1.7 (1.00,2.55)*
Irrigation	No	223 (71.3)	94 (28.7)	1	1
	Yes	40 (95.2)	2 (4.8)	0.12 (0.03, 0.52)	0.3 (0.88, 1.89)
DDS	>= 4	38 (92.7)	3 (7.3)	1	1
	< 4	404 (71)	165 (29)	5.2 (1.57, 16.99)	1.2 (0.28,4.43)
		Thinness			
		No	Yes		
Child sex	Male	366 (94.3)	22 (5.7)	1	1
	Female	195 (87.8)	27 (12.2)	2.3 (1.27,4.15)	1.8 (1.02, 3.48)*
Illness experienced	No	516 (94.3)	31 (5.7)	1	1
	Yes	45 (71.4)	18 (28.6)	6.6 (3.4, 12.82)	5.9 (3.04, 11.56)***
		Wasting			
		No	Yes		
HH sex	Male	233 (90)	26 (10)	1	1
	Female	284 (80.9)	67 (19.1)	2.1 (1.30,3.43)	1.5 (0.99, 2.48)
HH age	18–30	28 (71.8)	11 (28.2)	1	1
	>= 31	489 (85.6)	82 (14.4)	0.4 (0.20,0.89)	0.4 (0.20,0.94)*
Land ownership	No	189 (78.4)	52 (21.6)	2.2 (1.40, 3.44)	1.6 (1.05,2.63)*
	Yes	328 (88.9)	41 (11.1)	1	1
		Excess weight			
		No	Yes		
Child age	10–14	145 (85.8)	24 (14.2)	1	1
	15–19	411 (93.2)	30 (6.8)	0.4 (0.25,0.78)	0.3 (0.18,0.64)**
HH sex	Male	217 (83.8)	42 (16.2)	5.4 (2.81,10.61)	4.8 (2.43,9.62)***
	Female	339 (96.6)	12 (3.4)	1	1
Family size	2–4	221 (88)	30 (12)	1.8 (1.07,3.32)	1.8 (1.01,3.37)*
	>= 5	335 (93.3)	24 (6.7)	1	1
Off-farm income	No	520 (93)	39 (7)	1	1
	Yes	36 (70.6)	15 (29.4)	5.5 (2.80,11.01)	4.5 (2.15,9.54)***

*p value >= 05; **p value < 0.001; ***p value < 0.0001.

## Discussion

### Socio-demographic context and food security

Kuyu woreda, despite its initial designation as a crop growth corridor, has experienced significant agricultural setbacks due to adverse biophysical factors.^(19)^ This has led to increased reliance on food aid, underscoring the region’s vulnerability. Limited adoption of improved agricultural technologies, attributed to small landholdings, credit constraints, affordability, and availability, has likely exacerbated yield disparities. While technology adoption can improve nutritional outcomes,^(20,21)^ financial limitations hinder implementation, even with extension services. As Adams et al.^(22)^ suggest, financial support is vital for maximising the benefits of technology adoption. The low irrigation utilisation (7.4%) further highlights infrastructural and financial barriers.

The observed low meal frequency, particularly regarding breakfast and supper consumption, is indicative of household food insecurity. This aligns with findings from Amoadu et al.,^(23)^ who linked low-income and parental employment to malnutrition. Aneley et al.^(24)^ demonstrated the association between meal frequency, breakfast consumption, and academic performance, emphasising the importance of monitoring adolescent dietary intake to prevent malnutrition and related diseases.^(25)^

While cereals, a primary energy source, are frequently consumed, the low consumption of root crops and animal protein suggests dietary limitations. Ethiopian legumes offer a valuable protein source, but overall dietary diversity remains a concern. The finding that 93% of households provided fewer than five food groups, compared to the recommended five or above.^(12)^ Highlights poor household diet quality and limited food access. This is particularly concerning given the established link between dietary diversity, academic performance,^(24,25)^ and nutritional status.^(26–29)^ Poor diet quality has also been associated with an increased risk of being overweight and obese.^(30)^

### Anthropometric findings and comparative context

The meta-analysis by Hailegebriel^(31)^ reported a 22% thinness/wasting prevalence in Ethiopian schoolchildren, with regional variations ranging from 9% to 38%. Our study’s findings are within this range. Similarly, underweight prevalence in Ethiopia varies widely (27.5% to 51%), with our overall prevalence of 25.4% falling within the lower end of this range. Compared to other Southern and Eastern African countries, our findings are also within the observed range (5.8% to 27.1%). However, some studies have reported much higher underweight levels (44%) in Western Ethiopia.^(32)^ The observed differences between our study and that of Weres et al.^(33)^ regarding the prevalence of underweight in males and younger adolescents highlight the variability in adolescent nutritional status across different regions.

Stunting prevalence in Ethiopia also exhibits regional variation (7.2% to 27.5%). Our findings align with the higher end of this range. The contrasting findings regarding stunting in late adolescent girls between our study and that of Tariku et al.^(34)^ suggest potential regional differences in stunting patterns.

The association between female sex, late adolescence, and overweight, as reported by other workers in Ethiopia,^(35,36)^ is partly consistent with our findings. While we observed a higher prevalence of overweight in older girls, we also found that younger boys were more likely to be overweight. The complex interplay between food insecurity and overweight/obesity, as highlighted by Biadgilign et al.,^(37)^ underscores the need for nuanced interpretations.

The high prevalence of multiple growth failures in our study is consistent with Destaw et al.^(11)^ but differs from Kuiti et al.^(38)^ in terms of the specific types of failures. The low coexistence of stunting and overweight, as observed in our study and reported by Ejike et al.^(39)^ and Al-Taiar et al.,^(40)^ suggests complex underlying mechanisms.

### Factors associated with undernutrition and divergent findings

The association between female-headed households and stunting, as observed in our study, is consistent with Ashagidigbi et al.^(41)^ but contrasts with Zemene et al.^(42)^ Similarly, the association between female sex and underweight/overweight varies across studies.^(36,43)^ The contrasting findings regarding late adolescence and overweight between our study and Mandefro et al.,^(35)^ as well as Worku et al.,^(44)^ highlight the need for further exploration of age-related factors. The association of off-farm income with overweight, despite evidence suggesting its positive impact on food security,^(45,46)^ warrants further investigation into potential confounding factors.

### Strengths and limitations of the study

This study effectively assessed adolescent nutritional status via comprehensive data and robust methods, yielding clear recommendations. However, its cross-sectional design limits the ability to establish causality and generalizability, and potential biases exist in dietary and maternal self-report data, despite mitigation efforts such as the use of standardised questionnaires and trained interviewers.

### Conclusion

The study found high undernutrition among school-going adolescents due to poor meal frequency and dietary diversity. Therefore, immediate implementation of a school feeding programme is crucial. Targeted interventions should prioritise schoolgirls. Establishing school clinics for health monitoring, supporting landless households with income generation, and providing dietary education are also essential for improving adolescent nutritional status.

## Data Availability

All data generated or analysed during this study are included in this manuscript.
